# An Ergonomic Golf Grip Leads to Lower Forearm Muscle Activity - A Prospective Case Series of 30 Right-Handed Amateur and Professional Golfers

**DOI:** 10.1186/s12891-024-07774-7

**Published:** 2024-08-26

**Authors:** Jan Moritz Bochnia, Sebastian Bockholt, Georg Gosheger, Christoph Theil, Kristian Nikolaus Schneider

**Affiliations:** Golf Clinic, Department of Orthopaedics and Tumor Orthopaedics, University Hospital Münster (UKM), Albert-Schweitzer-Campus 1, 48149 Münster, Germany

**Keywords:** Epicondylitis, Ergonomic, EMG, Golf, Injuries, Overuse

## Abstract

**Background:**

The elbow is a common site for overuse injuries in golfers. Tendinopathies, such as medial and lateral epicondylitis, are frequently diagnosed in amateur and professional golfers. The aim of our study was to determine the effect of an ergonomic golf grip on forearm muscle activity during the five phases of the golf swing.

**Methods:**

Thirty right-handed golfers with a mean age of 32 years (range, 18–70 years) and a mean handicap of 15 (range, 0–43) performed 10 golf swings with a standard and ergonomic golf grip respectively. The mean and maximum muscle activity of the Musculus (M.) extensor carpi radialis brevis (ECRB), M. flexor carpi ulnaris (FCU), M. pronator teres (PT) and M. biceps brachii (BB) of the lead and trail arms were assessed during the five phases of the golf swing using surface electromyography (EMG). Subgroup analyses were performed regarding sex, playing ability (handicap < 10 vs. ≥10), weekly playing time (≤ 5 h, 5–20 h, > 20 h) and preexisting elbow pain during golfing (VAS < 2 vs. VAS ≥ 2). Significance was set at *p* < 0.05.

**Results:**

An ergonomic golf grip resulted in a reduction in muscle activity in at least one but up to three consecutive phases of the golf swing for the ECRB, FCU and PT of the lead arm and for the PT of the trail arm. Amateurs, a playing time < 20 h per week and golfers without preexisting elbow pain were factors that were associated with greater reductions in muscle activity.

**Conclusion:**

Forearm muscle activity can be decreased using an ergonomic golf grip, indicating the possible role of an ergonomic golf grip as a preventive measure against overuse syndromes such as medial and lateral epicondylitis.

**Trial registration number:**

This study was retrospectively registered at the German Clinical Trials Register DRKS-ID: DRKS00033732 (01/03/2024).

**Supplementary Information:**

The online version contains supplementary material available at 10.1186/s12891-024-07774-7.

## Background

Golf is a sport that is played all over the world. The sport is gaining increasing popularity, evidenced by the 10% increase in golfers in the United States from 22.4 million in 2012 to 25.6 million in 2022 [1]. Although golf is generally recognized as a low-injury sport that can be played regardless of age, the annual injury incidence rate is 15.8 per 100 golfers [2]. Out of all injuries reported by professional golfers, 10% were elbow injuries, while the rate was 24.9% among amateur golfers [3]. A common site for injury is the elbow, and overuse and poor swing mechanics are the predominant causes of medial and lateral epicondylitis [3–5]. Evidence proves that anomalous muscle activation of the forearm muscle can lead to muscle fatigue, which contributes to the development of elbow tendinopathies [6, 7]. Lateral epicondylitis is a typical overuse injury in golf, and for right-handed golfers, lateral epicondylitis commonly affects the lead arm, contrary to medial epicondylitis, which develops more frequently on the trail arm [8, 9]. According to prior research that measured forces acting on different joints involved in the mechanics of the golf swing, and studies that elaborated on muscle activity during the golf swing, the phases including ball contact, which are defined as acceleration and early follow-through by Jobe et al., are the most crucial parts of the golf swing concerning injuries to the forearm muscles and elbow joint [10–12]. Medial epicondylitis is an overuse tendinopathy at the origin of the forearm flexors [13]. As coming over the top and hitting “larger divots” could impede the pronation of the trail arm and cause injuries, the M. pronator teres is one of the muscles of interest [7]. Previous research has used electromyography (EMG) to evaluate differences between amateur and professional golfers, the impact of grip different grip sizes and commercial golf gloves on forearm muscle activity [14–16]. Forearm muscle activities have previously shown not to be affected by grip sizes or by the use of a commercial golf glove [15, 16]. However, forearm muscle activities did show differences between amateur and professional golfers: During the forward swing in the PT of the trail arm, the amateur golfers had significantly greater PT muscle activity than did the professional golfers. In contrast, in the acceleration phase in the PT of the lead arm, professional golfers had significantly greater PT activity than did the amateur golfers [14].

Thus the aim of our study was to investigate whether playing golf with an ergonomic golf grip, compared to playing golf with a standard golf grip, affects the forearm muscle activity monitored by surface EMG in adult right-handed male and female golfers, looking at the mean and maximum values of each phase of the golf swing for each muscle tested. We hypothesized that an ergonomic golf grip could reduce muscle activity in the forearm and elbow spanning muscles during the five phases of the golf swing. In addition, subgroup analyses were performed to identify beneficial and limiting factors for increased muscle activity in a large cohort of golfers.

## Methods

### Participants

Thirty right-handed golfers with a mean age of 32 years (range, 18–70 years) and a mean handicap of 15 (range, 0–43.4; handicaps were calculated by the World Handicap System) were recruited from a local country club and grouped according to sex, handicap, weekly playing time and elbow pain (Table 1). The study was approved by the local ethics committee (Ärztekammer Westfalen-Lippe. Reference number: 2021-696-f-S) and was performed in accordance with the Declaration of Helsinki (1964). Written informed consent was obtained from all participating golfers.

**Table 1:** Demographics, playing and clinical details

Variable	*n*	(%)
Sex		
Male	20	(66)
Female	10	(33)
Playing ability		
Amateur (HCP > 10)	17	(57)
Professional (HCP ≤ 10)	13	(43)
Time spent playing golf per week		
≤5 h	10	(33)
5–20 h	13	(43)
>20 h	7	(23)
Elbow pain during golfing		
Golfers with elbow pain, VAS ≥ 2	5	(17)
Golfers without elbow pain, VAS < 2	25	(83)

HCP: handicap, h: hours, VAS: visual analogue scale

### Golfing

After completing individual warm-ups, which included stretching, doing practice swings, and hitting golf balls with their own golf clubs, all golfers performed 20 golf swings hitting a Titleist Pro V1 golf ball (Acushnet Company, Fairhaven, USA) from a standard golf matt. The first 10 golf swings were performed with a standard golf grip, and the latter 10 were performed with an ergonomic golf grip. Only the last five golf swings of each set were used for further analysis, and the first five swings were used for calibration and for the golfers to get used to the experimental setting.

**Fig. 1:**
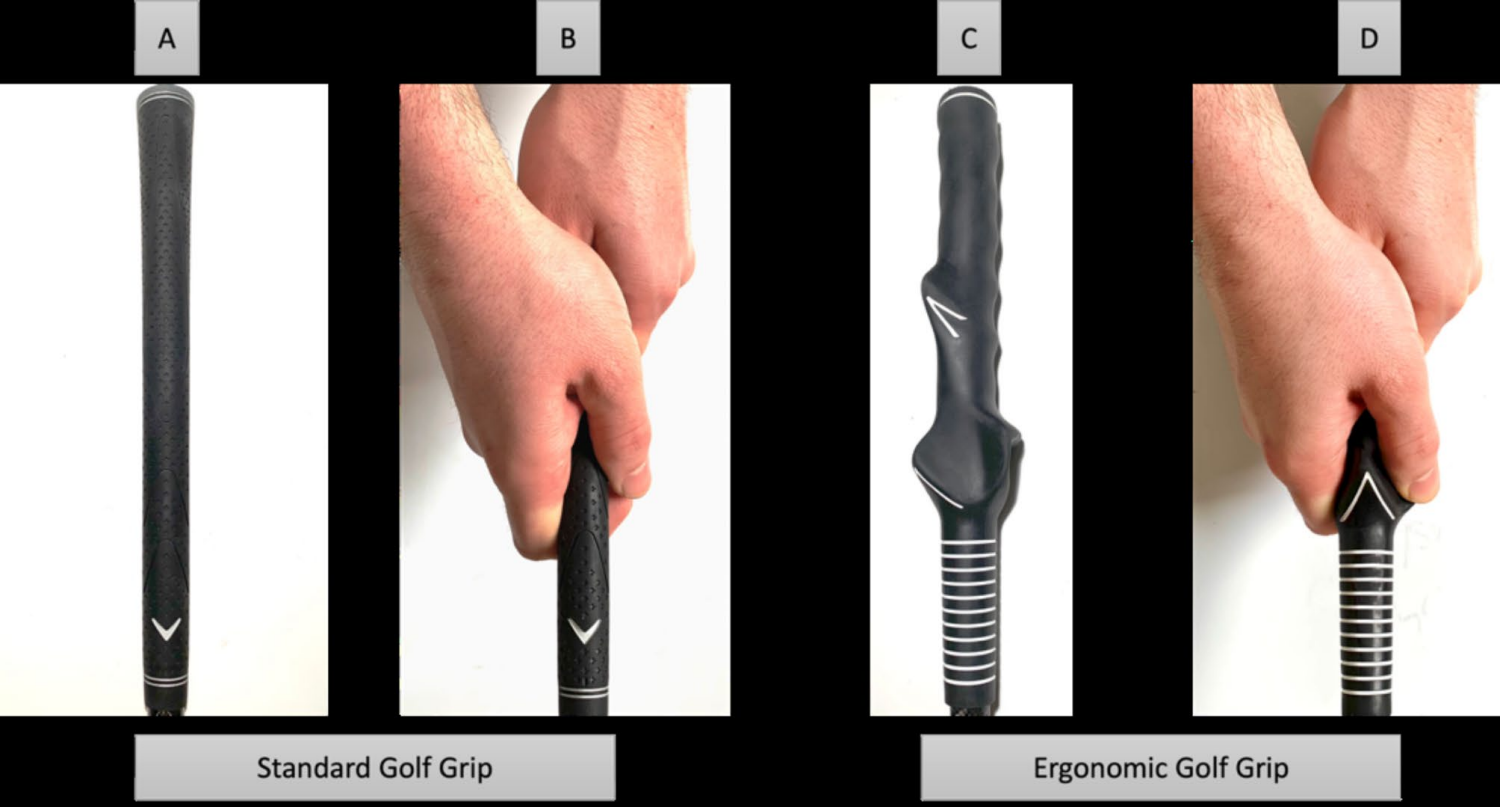
**A**–**D** The standard golf grip (Fig. **A** + **B**) and the ergonomic golf grip (Fig. **C** + **D**)

All golf swings were performed with a 7-iron (Callaway Golf, Carlsbad, USA), as this is the middle iron in a standard set of golf clubs [14–17]. For female golfers, a Callaway Rogue ST MAX OS Lite (Callaway Golf, Carlsbad, USA) with a ladies’ flex shaft (True Temper Sports, Memphis, USA) was used, and for male golfers, the corresponding Callaway Rogue ST MAX (Callaway Golf, Carlsbad, USA) with a regular flex shaft (Mitsubishi Chemical America, Carlsbad, USA) was used. While one golf club was fitted with a standard Golf Pride golf grip (Golf Pride, Pinehurst, USA), the other club was fitted with an ergonomic golf grip (Lamkin Training Grip, Lamkin Corporation, San Diego, USA; Fig. 1A-D).

### Electromyography (EMG)

**Fig. 2:**
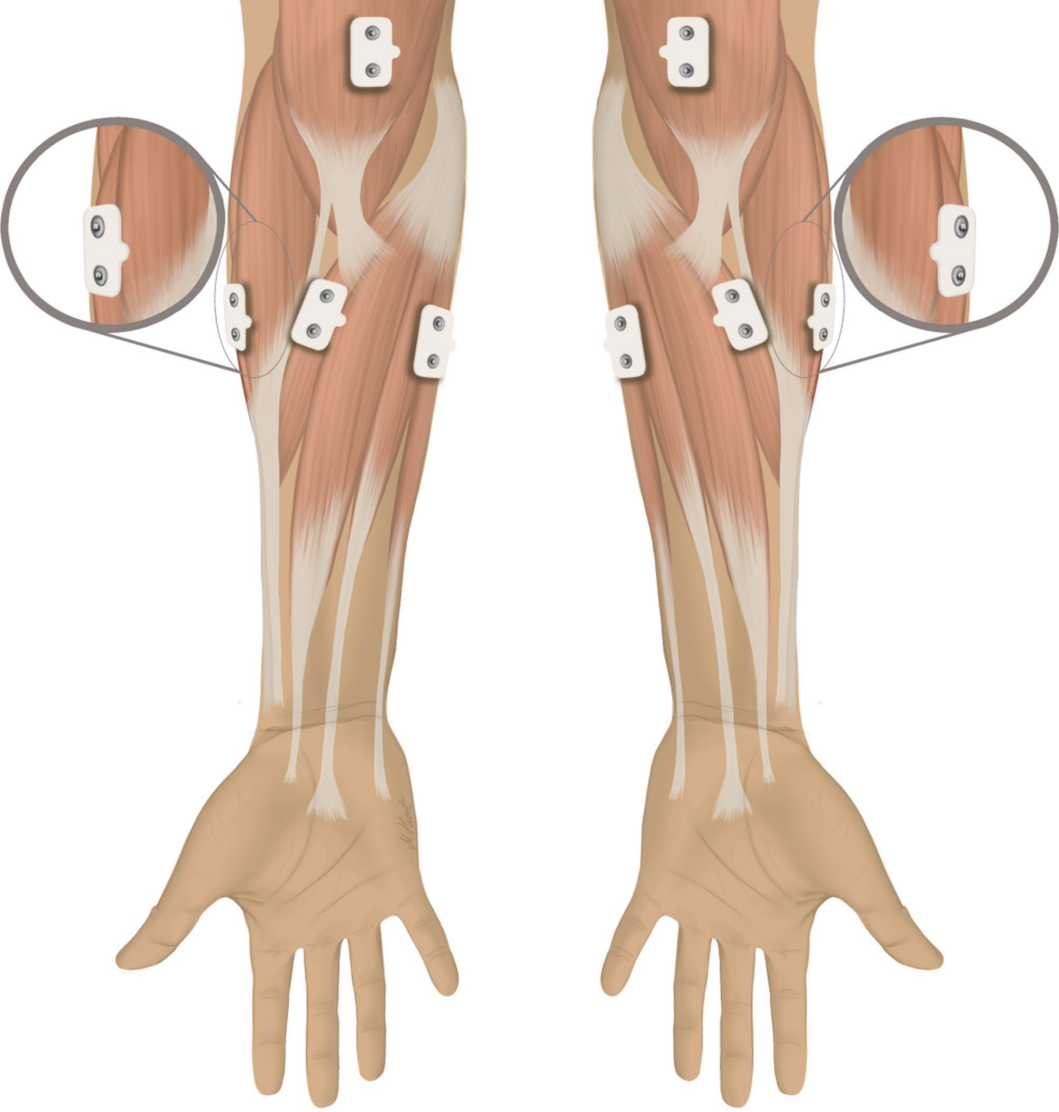
Anatomical drawing of the placement of surface EMG electrodes on the forearm muscles and M. biceps brachii; EMG: electromyography

Muscle activity was measured using surface EMG (MES9000; Myotronics-Noromed, Kent, USA) and a preamplifier (Myotronics-Noromed, Kent, USA). Bipolar silver chloride electrodes (Myotronics-Noromed, Kent, USA) with an interelectrode distance of 22 ± 1 mm were used. Muscle activity patterns measured in microvolt were assessed every 12.2 ms. The EMG signals were bandpass filtered at 50 Hz. After skin preparation, superficial electrodes were placed over the M. extensor carpi radialis brevis (ECRB), M. flexor carpi ulnaris (FCU), M. pronator teres (PT) and M. biceps brachii (BB) for the lead arm (the left arm in right-handed golfers) and the trail arm (the right arm in right-handed golfers; Fig. 2).

**Fig. 3:**
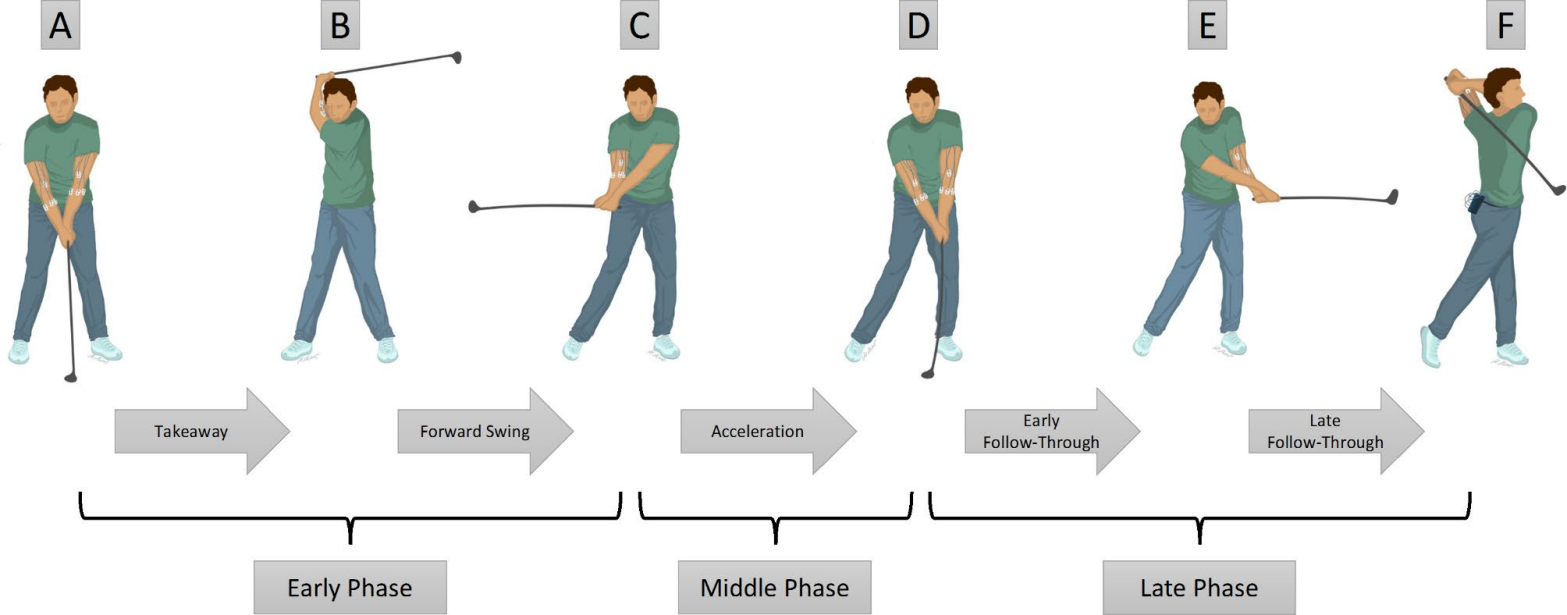
Five phases of the golf swing

The electrodes were connected to leads and united in the preamplifier. The preamplifier was fixed to the golfers’ back pocket, and EMG signals were transmitted at 240 to 1400 Hz to the EMG base station. All leads were long enough and were placed through the golfers’ shirts to minimize effects on the performed golf swing. All EMG signals were recorded with a digital video camera (Fujifilm X-T30, Fujifilm, Minato, Japan) positioned 2.0 m in front of the golfers to record the swing motion and EMG signals simultaneously. For further analysis, the golf swings were divided into five phases according to Jobe et al. (Fig. 3) [10]. The EMG data were synchronized to the video frames during each phase of the golf swing. Points indicating the start of each new phase were marked both in the video and in the Excel data. After the determination of all phases, the mean and maximum muscle activity of the respective muscles of the lead and trail arms were specified.

### Five phases of the golf swing

Jobe et al. defined the five phases of the golf swing as the *takeaway* from set up (A, Fig. 3) to the top of the backswing (B). The *forward swing* from the top of the backswing (B) to the horizontal position of the club on the back of a right-handed golfer (C). The *acceleration* from holding the golf club in the horizontal position on the back of a right-handed golfer (C) to ball contact (D). The *early follow-through* from ball contact (D) to club in the horizontal position in the front of a right-handed golfer (E) and the *late follow-through* from club in the horizontal position in the front of a right-handed golfer (E) to finish position (F) [10]. The *early phase* was defined as the takeaway and the forward swing; the *middle phase* was defined as the acceleration phase, and the *late phase* was defined as the early and late follow-through.

### Statistical analysis

An a priori power analysis was performed to identify the minimum number of participants required for assessing differences in muscle activity with the planned study setup. EMG data were collected in Microsoft Excel version 16.75.2 (Microsoft Corp., Redmond, WA, USA), and statistical analyses were performed with IBM SPSS Statistics version 28.0.0.0 (IBM Corp., Armonk, NY, USA). The data distribution was determined using the Kolmogorov-Smirnov test. For all nonparametric values, the mean and its respective range are given. Subgroup analyses were performed regarding sex, playing ability (handicap < 10 vs. ≥10), time spent playing golf per week (≤ 5 h, 5–20 h, > 20 h) and self-reported preexisting elbow pain during golfing, as stated on a VAS (VAS < 2 vs. VAS ≥ 2). Subgroup analyses were performed using a paired Wilcoxon signed rank test. All p values were two-sided, and the significance was set at *p* < 0.05.

## Results

Overall, when comparing an ergonomic to a standard golf grip, a reduced mean and maximum muscle activity was observed in 22.5% of all measurements during all five phases of the golf swing in the lead arm and in 15% in the trail arm (Table 2). Regarding the early phases of the golf swing, a reduction in mean and maximum muscle activity was recorded in 10% of all measurements in the lead arm and in 5% in the trail arm. Regarding the middle phase of the golf swing, we found a reduced muscle activity in 2.5% of all measurements in the lead arm and 5% in the trail arm. Regarding the late phase of the golf swing, 10% of all measurements in the lead arm and 5% in trail arm were lower compared to the standard grip. Increased muscle activity with an ergonomic golf grip was observed for 2.5% of all trail arm measurements and for no measurement of the lead arm.

**Table 2:** Results of the total cohort

Muscle	Takeaway		Forward swing		Acceleration		Early follow-through		Late follow-through	
	Mean	Max.	Mean	Max.	Mean	Max.	Mean	Max.	Mean	Max.
**Lead Arm**										
**ECRB**										
Standard	65.3	108.1	48.2	63.5	55	67.5	142.2	184.8	130.6	266.9
Ergonomic	64.6	112	44	65.1	61	72.2	117.4	159.2	127	289.8
* P-Value*	0.045*	0.024*	*0.845*	*0.428*	*0.719*	*0.558*	*0.28*	*0.09*	*0.614*	*0.417*
**FCU**										
Standard	76.6	134.4	123	157.4	115.4	137.5	138.5	173.1	120.2	255.3
Ergonomic	74.9	136.4	107.6	144.2	107.5	123.3	129.3	163.2	113.1	212.5
* P-Value*	*0.417*	*0.192*	*0.171*	*0.086*	*0.09*	0.043*	0.041*	0.006*	0.001*	0.01*
**PT**										
Standard	58.1	107.5	51.7	65.5	54.2	59.3	88.3	130.4	124	235.4
Ergonomic	54	93.7	53	67.3	54.5	58.8	88.5	112.4	118.7	253.4
* P-Value*	0.006*	0.017*	*0.53*	*0.504*	*0.861*	*1*	*0.704*	*0.289*	*0.478*	*0.371*
**BB**										
Standard	13.9	25.4	21.6	29.6	34.9	43.4	77.8	142	155.7	276
Ergonomic	14.1	31.3	20.5	30.4	35.7	49.5	86.3	149.9	163.9	277.6
* P-Value*	*0.465*	*0.294*	*0.206*	*0.417*	*0.057*	*0.159*	*0.441*	*0.688*	*0.845*	*0.309*
**Trail Arm**										
**ECRB**										
Standard	98.5	252.4	84	127.3	48.4	53.9	60.4	102.9	129.8	274.4
Ergonomic	97.4	240.1	85.1	141	49.7	56.9	59.2	81.4	120.1	273
* P-Value*	*0.926*	*0.861*	*0.766*	*0.614*	*0.829*	*0.797*	*0.371*	*0.185*	*0.153*	*0.271*
**FCU**										
Standard	61.2	98.9	68.7	97	107	124.3	201.4	244.4	158	330.4
Ergonomic	57.4	82.1	64	83.4	96.8	114.8	162.8	206.5	148.5	323.5
* P-Value*	*0.829*	*0.861*	*0.36*	*0.229*	*0.349*	*0.237*	*0.131*	*0.054*	*0.237*	*0.943*
**PT**										
Standard	48.6	120.7	39.3	57.5	41.3	52.6	91.3	131.9	105.1	245.6
Ergonomic	49.6	105.5	37.7	52.9	38.7	47.7	79.9	117	100	244.4
* P-Value*	*0.943*	*0.845*	0.006*	0.035*	0.021*	0.003*	0.007*	0.001*	*0.106*	*0.318*
**BB**										
Standard	40.1	98.1	38.2	57.3	41.7	47.7	66.8	137.6	125.7	300.5
Ergonomic	37.4	95.2	35,0.7	53.2	40	43.5	73.8	118.4	131.5	326.2
* P-Value*	*0.185*	*0.544*	*0.894*	*0.237*	*0.371*	*0.453*	*0.877*	*0.202*	*0.734*	0.047**

Mean and maximum µV of assessed muscle activity during the five phases of the golf swing

Statistically significant p-values are shown in bold, * indicates a statistically significant decrease in muscle activity with the ergonomic grip, ** indicates a statistically significant increase in muscle activity with the ergonomic grip

VAS: Visual analogue scale

### Lead arm muscle activation

In the lead arm lower mean and maximum muscle activities were found in the ECRB, FCU and PT, while no statistically significant changes in muscle activity were found for the BB (Table 2).

For the ECRB we found an increase in the mean and maximum muscle activity in the Takeaway, while in no other phase a significant change was detected (Table 2).

For the FCU, a reduction in muscle activity was observed with an ergonomic golf grip for three consecutive phases of the golf swing: maximum muscle activity (*p* = 0.043) during the acceleration, mean (*p* = 0.041) and maximum (*p* = 0.006) muscle activity during the early follow-through and mean (*p* = 0.001) and maximum (*p* = 0.01) muscle activity during the late follow-through.

For the PT, a reduction in the mean (*p* = 0.006) and maximum (*p* = 0.017) muscle activity was observed during the takeaway.

No increased or decreased muscle activities were observed for the BB (Table 2).

### Trail arm muscle activation

In the trail arm lower mean and maximum muscle activities were found in the PT, higher maximum activity was detected for one phase in the BB, while there were no changes in muscle activity was observed for the ECRB and FCU (Table 2).

For the PT, reductions in the mean (*p* = 0.006) and maximum (*p* = 0.035) muscle activity during the forward swing, acceleration (mean *p* = 0.021; max. *p* = 0.003) and early follow-through (mean *p* = 0.007; max. *p* < 0.001) were observed.

The only increase in muscle activity with an ergonomic golf grip was observed for the BB in the trail arm with an increased maximum muscle activity during the late follow-through (*p* = 0.047).

### Beneficial and limiting factors

In both female and male golfers, a reduction in muscle activity was observed with the use of an ergonomic grip, but the test results differed significantly between the sexes regarding the phase of the golf swing (Appendix Table 1). Overall, more statistically significant reductions in muscle activity with the ergonomic grip were found in amateurs than in professional golfers, and both amateurs and professional golfers showed overall lower ECRB muscle activity. The phases of the golf swing as well as the respective arm differed: mean muscle activity (*p* = 0.049) during the takeaway in the ECRB of the lead arm for amateurs and for professional golfers maximum muscle activity (*p* = 0.046) during the forward swing in the ECRB of the trail arm. Golfers who play 20 h of golf per week or less showed greater reduction in muscle activity with an ergonomic grip than golfers who play more than 20 h per week. Golfers without elbow pain overall had more reduced muscle activity with the ergonomic grip than golfers with preexisting elbow pain. Golfers without preexisting elbow pain, lower muscle activities in the PT and FCU of the lead arm and in the PT of the trail arm were observed with the ergonomic golf grip while golfers with preexisting elbow pain, lower FCU, PT and BB muscle activities in the lead arm and for the BB in the trail arm were observed. Higher muscle activities with the ergonomic golf grip were found for golfers without preexisting elbow pain in the maximum value of the late follow-through in the BB of the trail arm (*p* = 0.016) and for golfers with elbow pain in the ECRB of the lead arm in the mean and maximum values of the forward swing (mean *p* = 0.043; max. *p* = 0.043).

## Discussion

This prospective case series aimed to understand how an ergonomic golf grip compared to a standard golf grip affects forearm muscle activity during the five phases of the golf swing. Considering the results of the surface EMG measurements, (1) an ergonomic golf grip leads to reduced muscle activity in the forearm muscles ECRB and FCU. (2) Golfers who use an ergonomic golf grip could experience reduced muscle activity of the forearm pronating muscle, the PT, while they may not experience the same effects on the elbow flexing muscle, the BB. (3) Remarkably, our subgroup analyses highlighted that greater reductions in muscle activity were observed among amateurs, golfers who play 20 h per week or less and golfers without preexisting elbow pain.

The elbow joint is one of the most common injury sites in golfers, who predominantly suffer from overuse injuries [2, 3]. We found that with an ergonomic golf grip, lower muscle activity was observed for the ECRB, the FCU and the PT of the lead arm and for the PT of the trail arm. For the ERCB, the mean and maximum muscle activity were reduced, especially during takeaway, and a previous study showed that holding the grip to tight in set-up position is a potential reason for elbow and wrist injuries. An ergonomic golf grip can help to reduce muscle activity and risk of injury during these crucial early phases of the golf swing [18].

The FCU of the lead arm, demonstrated reduced muscle activity during the last three phases of the golf swing, with the greatest reductions in muscle activity being measured in the late follow-through. Previous studies measured the forces acting on different joints during the golf swing and found that both wrist joints are exposed to rapid accelerations and abrupt decelerations, with the highest forces being measured just before and just after hitting the golf ball [11, 12]. Because golfers typically hit the ground with a golf club just after impact with the ball, there is an abrupt, sharp deceleration in wrist flexion that can create valgus forces on the elbow joint. Physiologically a combination of osseous arrangement and the activation of the flexor-pronator mass are assisting the medial ulnar collateral ligament in acting against these valgus forces. The M. flexor carpi radialis, M. flexor carpi ulnaris, and M. pronator teres have all been shown to be active on EMG testing when they resist valgus moments. Thus, by reducing the muscle activity of the forearm flexors, overuse injuries to the medial epicondyle may be avoided, as the flexors contribute to the dynamic stabilization of the elbow and can counteract the valgus forces [5, 19–25]. We found that the FCU muscle activity in the lead arm was lower with the ergonomic golf grip during these phases of the golf swing, including contact with the golf ball. Conversely, the trail arm is the typical site for medial epicondylitis; despite not reaching statistical significance, there was a tendency for a reduction in muscle activity in the FCU with an ergonomic golf grip, as muscle activity was lower during all phases for all tested golfers. Combining the results of the FCU and the PT of the trail arm, it appears that the ergonomic grip leads to reduced muscle activity in the muscles attached to the common flexor tendon, contributing to elbow stabilization.

The greatest reductions in muscle activity were found for the golfers’ PT of the trail arm. Pronation of the trail arm takes place mainly during the early follow-through just after the golf ball is struck. Coming over the top and hitting “larger divots” could impede the pronation of the trail arm and cause injuries [7]. The findings of our study showed that PT muscle activity decreases precisely during the phases involving ball striking and pronating the forearm. Pronation of the lead arm takes place at the takeaway as the golf club moves to the right of right-handed golfers. Even though PT injuries do not typically occur during takeaway it could be an efficient way to warm up by not forcing full muscle activity during the first swings. Interestingly, the muscle activity of the BB, the primary forearm supinator, increased continuously until the end of the golf swing, with even higher muscle activity being measured using an ergonomic grip compared to a standard grip. Previous EMG studies on golfers evaluating forearm muscle activity have not included the BB [14–16]. Our study aimed to investigate both a pronator and a supinator muscle of the forearm. The data from our research could be valuable for future studies seeking to understand the pathologies of the BB and its tendons resulting from playing golf.

Several beneficial and limiting factors for the use of an ergonomic golf grip were identified. Previous studies have shown that amateur golfers have a greater incidence of elbow injuries than professional golfers [3, 7]. In our cohort, amateur golfers showed a greater reduction in muscle activity than professional golfers. Despite being more experienced and equipped with a better gripping technique, reductions in PT muscle activity in the trail arm of professional golfers during acceleration and early follow-through—the phases of the golf swing in which most elbow injuries occur—potentially indicate a role for the ergonomic golf grip not only for amateurs but also for professional golfers [7]. Earlier investigations have shown that the prevalence of elbow injuries is similar for both female and male golfers [3, 7, 8]. This finding is in line with the muscle activity observed in our cohort, which did not significantly differ between the sexes. Furthermore, prior research has shown that the more golfers play, the more injuries they suffer. However, the findings of our study indicate that an ergonomic grip is especially beneficial for amateur golfers who do not spend as much time per week playing golf as professionals are [2].

A pivotal question of our study was to determine whether an ergonomic golf grip could be a potential measure for reducing forearm muscle activity in golfers suffering from elbow pain during golfing. Sorbie et al. examined forearm muscle activity using different grip sizes while playing golf and playing golf with and without a commercial golf glove. Neither study identified any preventive or therapeutic measures for the devices that were tested [15, 16]. However, our study showed that the ergonomic golf grip decreases muscle activity, especially in golfers who have no preexisting elbow pain. Thus, an ergonomic golf grip may be a more preventive measure than a therapeutic measure for individuals suffering from preexisting elbow pain.

### Limitations

Several limitations must be acknowledged. Since the golf swing is one of the most complex sequences of movements in sports, it is challenging to repeat identical golf swings for the assessment of muscle activity [11, 26]. This is especially true for higher handicap golfers, in whom the variations in golf swings may be greater than those for professional golfers, possibly affecting the results and findings of this study. To reduce these variations, we increased the cohort of golfers, performed a standardized study protocol, assessed five golf swings per individual golfer and used mean and maximum values across these five golf swings to further reduce outlier values.

We are aware that the use of surface EMG is not as accurate as fine-wire EMG for assessing muscle activity [27]. However, as we have assessed muscle activity during the dynamics of a golf swing with rapid and complex muscle contractions, surface EMG might be less prone to potential injuries such as bruising, haematomas or even infections, which have previously been described for fine-wire EMG [28].

Currently, there are rules in golf, stating that an ergonomic grip cannot be used in tournament rounds (R&A Rule 4.3 Use of Equipment Sect. 6 Stretching Devices and Training or Swing Aids, USGA Rule 4.3 Use of Equipment Sect. 6 Stretching Devices and Training or Swing Aids). The USGA and the R&A already have a rule for the use of equipment for golfers with medical reasons (The R&A Rule 4.3b, USGA Rule 4.3b); however, a committee must decide whether the use of equipment could give golfers any advantage over other players. As the grip is one of the most important parts of the execution of a golf swing, it will not be easy to convince the committee to use an ergonomic golf grip. However, the findings of our study suggest that an ergonomic golf grip helps to reduce muscle activity, which is associated with overuse, as such, its usage should be discussed for well-selected subsets of golfers suffering overuse injuries [29, 30].

Regarding subgroup analyses, we have to acknowledge, that not all subgroups were equally big so that future studies should consider similar group sizes to ensure better comparability (Table 1).

In addition, for this study protocol, the use of the ergonomic and standard golf grip has not been randomized. Future studies could use randomization to further strengthen findings and conclusions.

We are aware that modern technology can provide extensive information regarding distance, clubhead speed, swing path, etc., and should be used in future studies. Nevertheless, in our study, we aimed for all participants and researchers involved in this project to focus on muscle activity. Future studies could examine the accuracy, length, fatigue, or even other parameters. A patient who can play golf without pain is likely willing to make some sacrifices in length or precision in his game.

## Conclusion

An ergonomic golf grip leads to a reduction in muscle activity in the forearm muscles ECRB and FCU and in the forearm rotating muscle PT of the lead arm and the PT of the trail arm, while no effect on the elbow flexing muscle BB was observed in the lead or trail arm. Amateurs, a playing time of less than 20 h per week and golfers without preexisting elbow pain were associated with greater reductions in muscle activity.

### Electronic supplementary material

Below is the link to the electronic supplementary material.

Supplementary Material 1

## Data Availability

The datasets used and analysed during the current study are available from the corresponding author on reasonable request.

## Abbreviations

BB: Musculus biceps brachii
ECRB: Musculus extensor carpi radialis brevis
EMG: Electromyography
FCU: Musculus flexor carpi ulnaris
HCP: Handicap
PT: Musculus pronator teres
VAS: Visual analogue scale
