# Supplementary material for: An Ergonomic Golf Grip Leads to Lower Forearm Muscle Activity - A Prospective Case Series of 30 Right-Handed Amateur and Professional Golfers
Source: BMC Musculoskelet Disord. 2024 Aug 26;25:668. doi: 10.1186/s12891-024-07774-7 (PMC11346012; doi:10.1186/s12891-024-07774-7)
Supplement: Supplementary file 1 — Supplementary Material 1 [file 12891_2024_7774_MOESM1_ESM.docx]

**Appendix**

**Table 1**

| Muscle  Grip | Takeaway | | Forward Swing | | Acceleration | | Early Follow-Through | | Late Follow-Through | |
| --- | --- | --- | --- | --- | --- | --- | --- | --- | --- | --- |
|  | Mean | Max. | Mean | Max. | Mean | Max. | Mean | Max. | Mean | Max. |
| **Lead Arm** | | | | | | | | | | |
| ECRB |  |  |  |  |  |  |  |  |  |  |
| Total cohort |  |  |  |  |  |  |  |  |  |  |
| Standard | 65,3 | 108,1 | 48,2 | 63,5 | 55 | 67,5 | 142,2 | 184,8 | 130,6 | 266,9 |
| Ergonomic | 64,6 | 112 | 44 | 65,1 | 61 | 72,2 | 117,4 | 159,2 | 127 | 289,8 |
| P-Value | **0,045*** | **0,024*** | 0,845 | 0,428 | 0,719 | 0,558 | 0,28 | 0,09 | 0,614 | 0,417 |
| Male |  |  |  |  |  |  |  |  |  |  |
| Standard | 52,4 | 104,1 | 55,1 | 69,7 | 65 | 96 | 158,3 | 216,3 | 142,8 | 304 |
| Ergonomic | 50,4 | 96,1 | 49,8 | 73,2 | 65,4 | 74,5 | 143,5 | 187,8 | 138,3 | 295,6 |
| P-Value | 0,247 | 0,093 | 1 | 0,455 | 0,502 | 0,433 | 0,191 | 0,067 | 0,391 | 1 |
| Female |  |  |  |  |  |  |  |  |  |  |
| Standard | 81 | 140,5 | 37,4 | 45,8 | 43,5 | 51,6 | 95,3 | 148,9 | 108,3 | 220,9 |
| Ergonomic | 83 | 134 | 40,1 | 46,6 | 45 | 59,9 | 103,1 | 141 | 118,7 | 233,2 |
| P-Value | 0,074 | 0,139 | 0,721 | 0,508 | 0,721 | 0,959 | 0,799 | 0,721 | 0,646 | 0,139 |
| Professionals |  |  |  |  |  |  |  |  |  |  |
| Standard | 62,5 | 104,8 | 54,6 | 67,4 | 59,6 | 93,3 | 143,6 | 183,9 | 106,8 | 246,5 |
| Ergonomic | 52,7 | 110,2 | 54 | 65,3 | 61,8 | 73,4 | 119,2 | 151,6 | 106,7 | 250,1 |
| P-Value | 0,463 | 0,196 | 0,65 | 0,279 | 0,279 | 0,196 | 0,311 | 0,311 | 0,345 | 0,133 |
| Amateurs |  |  |  |  |  |  |  |  |  |  |
| Standard | 70,8 | 111,5 | 40,6 | 59,4 | 51,8 | 54,9 | 118,7 | 185,7 | 143,9 | 299,1 |
| Ergonomic | 70,2 | 121,7 | 40,6 | 65 | 60,2 | 71 | 115,5 | 166,8 | 140,1 | 301,7 |
| P-Value | **0,049*** | 0,102 | 0,981 | 0,619 | 0,554 | 0,687 | 0,554 | 0,266 | 0,309 | 0,653 |
| Playing Time ≤5h |  |  |  |  |  |  |  |  |  |  |
| Standard | 59 | 103,5 | 56,1 | 69,4 | 76 | 110,5 | 204 | 243,3 | 146,3 | 304 |
| Ergonomic | 66 | 116,6 | 53,4 | 70,4 | 85 | 114 | 167,7 | 208,4 | 136,3 | 296,3 |
| P-Value | 0,386 | 0,445 | 0,878 | 0,333 | 0,074 | 0,114 | 0,059 | **0,047*** | 0,285 | 0,721 |
| Playing Time 5-20h |  |  |  |  |  |  |  |  |  |  |
| Standard | 70,8 | 135,2 | 40,6 | 59,4 | 44,1 | 50,3 | 89,7 | 136,3 | 129,4 | 291,7 |
| Ergonomic | 65,9 | 121,7 | 40,6 | 70,5 | 60,2 | 68,1 | 108 | 126,9 | 115,8 | 290 |
| P-Value | **0,016*** | **0,039*** | 0,65 | 0,463 | 0,463 | 0,552 | 0,972 | 0,382 | 0,422 | 0,507 |
| Playing Time >20h |  |  |  |  |  |  |  |  |  |  |
| Standard | 65,8 | 104,8 | 46,9 | 60,3 | 58,9 | 72,1 | 140,9 | 183,9 | 92 | 187,9 |
| Ergonomic | 63,4 | 110,2 | 45,2 | 53,5 | 47,5 | 73,4 | 139,7 | 192 | 106,7 | 250,1 |
| P-Value | 0,398 | 0,499 | 0,612 | 0,176 | 0,735 | 0,612 | 0,866 | 0,866 | 0,091 | 0,063 |
| VAS <2 |  |  |  |  |  |  |  |  |  |  |
| Standard | 65,8 | 111,5 | 54,6 | 72 | 59,6 | 93,3 | 143,6 | 183,9 | 129,4 | 287,2 |
| Ergonomic | 65,9 | 112,2 | 54 | 72,6 | 68,9 | 75,6 | 119,2 | 166,8 | 132,4 | 290 |
| P-Value | 0,104 | 0,054 | 0,677 | 0,115 | 0,443 | 0,276 | 0,211 | 0,115 | 0,638 | 0,427 |
| VAS ≥2 |  |  |  |  |  |  |  |  |  |  |
| Standard | 37,3 | 69,8 | 19,6 | 23 | 28,2 | 36,7 | 118,7 | 185,7 | 138,8 | 246,3 |
| Ergonomic | 37,3 | 77,9 | 20,5 | 30,4 | 39,7 | 62,1 | 115,5 | 146,4 | 121,5 | 240,5 |
|  | 0,225 | 0,345 | **0,043**** | **0,043**** | 0,5 | 0,225 | 0,893 | 0,5 | 0,893 | 0,686 |
| **Trail Arm** | | | | | | | | | | |
| ECRB |  |  |  |  |  |  |  |  |  |  |
| Total cohort |  |  |  |  |  |  |  |  |  |  |
| Standard | 98,5 | 252,4 | 84 | 127,3 | 48,4 | 53,9 | 60,4 | 102,9 | 129,8 | 274,4 |
| Ergonomic | 97,4 | 240,1 | 85,1 | 141 | 49,7 | 56,9 | 59,2 | 81,4 | 120,1 | 273 |
| P-Value  Male | 0,926 | 0,861 | 0,766 | 0,614 | 0,829 | 0,797 | 0,371 | 0,185 | 0,153 | 0,271 |
|  |  |  |  |  |  |  |  |  |  |  |
| Standard | 101,9 | 275,5 | 115,1 | 159,2 | 59,4 | 73 | 76,1 | 108,9 | 147,8 | 301,1 |
| Ergonomic | 100,4 | 267,3 | 100,9 | 166 | 66 | 83,6 | 65,7 | 97,8 | 146,5 | 298,7 |
| P-Value  Female | 0,765 | 0,823 | 0,455 | 0,37 | 0,97 | 0,794 | 0,478 | 0,37 | 0,351 | 0,247 |
|  |  |  |  |  |  |  |  |  |  |  |
| Standard | 83,6 | 164 | 44,4 | 70,6 | 34,9 | 38,8 | 43 | 63 | 88,8 | 175,4 |
| Ergonomic | 84,9 | 158,8 | 46,7 | 67,7 | 34,6 | 38,3 | 51,2 | 77,4 | 94,7 | 183,9 |
| P-Value  Professionals | 0,575 | 0,285 | 0,575 | 0,799 | 0,646 | 0,445 | 0,508 | 0,444 | 0,285 | 0,878 |
|  |  |  |  |  |  |  |  |  |  |  |
| Standard | 91,9 | 207 | 112,6 | 149,3 | 58,2 | 65,2 | 77,1 | 102,7 | 127,6 | 254,1 |
| Ergonomic | 93,1 | 212,1 | 99,3 | 120,3 | 61,2 | 83,6 | 61,9 | 99 | 154,8 | 310,6 |
| P-Value  Amateurs | 0,249 | 0,279 | 0,116 | **0,046*** | 0,917 | 0,861 | 0,65 | 0,507 | 0,701 | 0,917 |
|  |  |  |  |  |  |  |  |  |  |  |
| Standard | 123,8 | 270 | 83,1 | 122,3 | 45,6 | 49,1 | 52,8 | 103,1 | 132 | 275,4 |
| Ergonomic | 119,7 | 259,6 | 80,6 | 142,6 | 49,4 | 54,8 | 57,8 | 78 | 109,5 | 268,5 |
| P-Value  Playing Time ≤5h | 0,309 | 0,463 | 0,356 | 0,381 | 0,586 | 0,407 | 0,435 | 0,193 | 0,068 | 0,124 |
|  |  |  |  |  |  |  |  |  |  |  |
| Standard | 99,5 | 221,7 | 89,1 | 133,3 | 41,6 | 44,4 | 60,4 | 111,3 | 133,1 | 279,9 |
| Ergonomic | 104,7 | 212,6 | 96,2 | 156,8 | 54,8 | 61,3 | 63,2 | 88,5 | 120,1 | 304,5 |
| P-Value  Playing Time 5-20h | 0,646 | 0,959 | 0,333 | 0,285 | 0,508 | 0,241 | **0,047*** | **0,022*** | 0,721 | 0,959 |
|  |  |  |  |  |  |  |  |  |  |  |
| Standard | 98,1 | 250,5 | 72,9 | 104 | 43,8 | 48,5 | 52,8 | 85,5 | 112,8 | 226,1 |
| Ergonomic | 100,1 | 249,7 | 61,3 | 80,6 | 39,2 | 42,6 | 57,7 | 79,3 | 109,1 | 200,7 |
| P-Value  Playing Time >20h | 0,701 | 0,552 | 0,173 | 0,311 | 0,807 | 0,807 | 0,701 | 0,65 | 0,087 | 0,152 |
|  |  |  |  |  |  |  |  |  |  |  |
| Standard | 91,9 | 254,3 | 120,5 | 182,6 | 62,1 | 83,1 | 98,6 | 162,7 | 127,6 | 304,8 |
| Ergonomic | 93,1 | 214,9 | 102,4 | 161 | 73 | 103,3 | 61,9 | 112 | 170 | 320,9 |
| P-Value  VAS <2 | 0,176 | 0,237 | 0,866 | 0,237 | 1 | 0,735 | 0,735 | 0,866 | 0,735 | 0,612 |
|  |  |  |  |  |  |  |  |  |  |  |
| Standard | 98,9 | 254,3 | 100,9 | 146,2 | 54,7 | 61,9 | 69 | 108 | 132 | 286,4 |
| Ergonomic | 100,1 | 240,8 | 102,4 | 161 | 59,6 | 63,5 | 60,3 | 83,5 | 130,7 | 277,5 |
| P-Value  VAS ≥2 | 0,657 | 0,619 | 0,737 | 0,677 | 0,968 | 0,968 | 0,276 | 0,174 | 0,201 | 0,276 |
|  |  |  |  |  |  |  |  |  |  |  |
| Standard | 82,4 | 123,8 | 23,8 | 29,3 | 16,1 | 18,9 | 35,5 | 69,8 | 85,6 | 248,4 |
| Ergonomic | 86,4 | 141,9 | 25,8 | 33,8 | 19,3 | 20,9 | 31,5 | 60,7 | 84,3 | 243,2 |
| P-Value | 0,5 | 0,5 | 0,5 | 0,5 | 0,138 | 0,08 | 0,893 | 0,893 | 0,345 | 0,686 |
| **Lead Arm** | | | | | | | | | | |
| FCU |  |  |  |  |  |  |  |  |  |  |
| Total cohort |  |  |  |  |  |  |  |  |  |  |
| Standard | 76,6 | 134,4 | 123 | 157,4 | 115,4 | 137,5 | 138,5 | 173,1 | 120,2 | 255,3 |
| Ergonomic | 74,9 | 136,4 | 107,6 | 144,2 | 107,5 | 123,3 | 129,3 | 163,2 | 113,1 | 212,5 |
| P-Value  Male | 0,417 | 0,192 | 0,171 | 0,086 | 0,09 | **0,043*** | **0,041*** | **0,006*** | **0,001*** | **0,01*** |
|  |  |  |  |  |  |  |  |  |  |  |
| Standard | 79,3 | 153,7 | 121 | 150,1 | 111,8 | 146,7 | 168,5 | 216,4 | 168,6 | 327,4 |
| Ergonomic | 78,4 | 160,2 | 101,8 | 127,8 | 107,5 | 123,3 | 147,6 | 171,8 | 142,4 | 285,6 |
| P-Value  Female | 0,823 | 0,332 | 0,232 | 0,263 | 0,263 | 0,126 | 0,156 | 0,086 | **0,014*** | **0,028*** |
|  |  |  |  |  |  |  |  |  |  |  |
| Standard | 72,3 | 128,5 | 125,2 | 159,6 | 122,9 | 128,2 | 129,6 | 166,8 | 117,7 | 191 |
| Ergonomic | 74,2 | 129,7 | 128,1 | 153 | 104,8 | 113,1 | 127,6 | 137 | 105,6 | 182,7 |
| P-Value  Professionals | 0,203 | 0,445 | 0,386 | 0,114 | 0,139 | 0,169 | 0,093 | **0,013*** | 0,059 | 0,169 |
|  |  |  |  |  |  |  |  |  |  |  |
| Standard | 80,7 | 171,1 | 94,2 | 136,7 | 136,3 | 149,7 | 192,8 | 261,4 | 147,9 | 383,1 |
| Ergonomic | 93,1 | 171,5 | 103,4 | 155,2 | 131,6 | 137,3 | 180,2 | 196,8 | 103,4 | 286,3 |
| P-Value  Amateurs | 0,65 | 0,422 | 0,463 | 0,345 | 0,311 | 0,221 | 0,152 | 0,221 | 0,087 | 0,116 |
|  |  |  |  |  |  |  |  |  |  |  |
| Standard | 69,3 | 130,2 | 130,3 | 162,9 | 107,9 | 125 | 130,1 | 167,6 | 120 | 217,3 |
| Ergonomic | 65,7 | 115 | 111,8 | 137,6 | 93,1 | 113,2 | 127,8 | 135,8 | 118,5 | 209,3 |
| P-Value  Playing Time ≤5h | 0,523 | 0,435 | 0,227 | 0,21 | 0,177 | 0,124 | 0,163 | **0,017*** | **0,006*** | **0,039*** |
|  |  |  |  |  |  |  |  |  |  |  |
| Standard | 68,8 | 135,8 | 107,8 | 144,3 | 100,8 | 110,5 | 149,5 | 185,8 | 129,5 | 284,9 |
| Ergonomic | 72,4 | 136,4 | 114,4 | 156,5 | 112,6 | 127,2 | 156,3 | 175,6 | 125,4 | 254,2 |
| P-Value  Playing Time 5-20h | 0,646 | 0,575 | 0,386 | 0,508 | 0,333 | 0,285 | 0,959 | 0,203 | **0,047*** | 0,139 |
|  |  |  |  |  |  |  |  |  |  |  |
| Standard | 78,1 | 130,3 | 140,9 | 203,3 | 127 | 153,1 | 146,3 | 174,7 | 120 | 185,3 |
| Ergonomic | 76,6 | 117,9 | 130,9 | 150,9 | 102,7 | 120,2 | 128,3 | 138 | 107,7 | 192 |
| P-Value  Playing Time >20h | 0,382 | 0,249 | 0,152 | **0,046*** | **0,019*** | **0,009*** | **0,019*** | **0,016*** | 0,055 | **0,039*** |
|  |  |  |  |  |  |  |  |  |  |  |
| Standard | 75,3 | 136,4 | 94,2 | 120,2 | 136,3 | 149,7 | 126,4 | 171,4 | 98,1 | 266,9 |
| Ergonomic | 97,1 | 160 | 64,5 | 111,3 | 131,6 | 137,3 | 124,8 | 163,1 | 93,3 | 186,4 |
| P-Value  VAS <2 | 0,31 | 0,128 | 0,176 | 0,128 | 0,128 | 0,128 | 0,31 | 0,612 | 0,091 | 0,398 |
|  |  |  |  |  |  |  |  |  |  |  |
| Standard | 78,1 | 144,2 | 125,9 | 162,9 | 129,4 | 149,7 | 130,7 | 171,4 | 120 | 243,8 |
| Ergonomic | 76,6 | 142,6 | 125,3 | 155,2 | 121,4 | 128,2 | 127,8 | 163,1 | 107,7 | 209,3 |
| P-Value  VAS ≥2 | 0,581 | 0,211 | 0,104 | **0,048*** | **0,045*** | **0,032*** | 0,093 | **0,026*** | **0,002*** | **0,014*** |
|  |  |  |  |  |  |  |  |  |  |  |
| Standard | 64,3 | 100,9 | 56,6 | 76,8 | 90,7 | 119,1 | 146,3 | 203,1 | 138,7 | 299,5 |
| Ergonomic | 58,5 | 115 | 56,1 | 99,9 | 81,7 | 113 | 130,3 | 163,3 | 142,2 | 286,3 |
| P-Value | 0,5 | 0,5 | 0,5 | 0,5 | 0,345 | 0,893 | 0,225 | **0,043*** | 0,345 | 0,5 |
| **Trail Arm** | | | | | | | | | | |
| FCU |  |  |  |  |  |  |  |  |  |  |
| Total cohort |  |  |  |  |  |  |  |  |  |  |
| Standard | 61,2 | 98,9 | 68,7 | 97 | 107 | 124,3 | 201,4 | 244,4 | 158 | 330,4 |
| Ergonomic | 57,4 | 82,1 | 64 | 83,4 | 96,8 | 114,8 | 162,8 | 206,5 | 148,5 | 323,5 |
| P-Value  Male | 0,829 | 0,861 | 0,36 | 0,229 | 0,349 | 0,237 | 0,131 | 0,054 | 0,237 | 0,943 |
|  |  |  |  |  |  |  |  |  |  |  |
| Standard | 64,8 | 102,5 | 74,1 | 96,8 | 103,1 | 123,4 | 206,7 | 275,8 | 161,6 | 364,1 |
| Ergonomic | 59,7 | 92,9 | 57,7 | 73,4 | 94,1 | 112,9 | 178,7 | 231,2 | 161,4 | 324,1 |
| P-Value  Female | 0,601 | 0,654 | 0,191 | 0,247 | 0,191 | 0,156 | 0,145 | 0,086 | 0,502 | 0,97 |
|  |  |  |  |  |  |  |  |  |  |  |
| Standard | 47,1 | 71,6 | 61,9 | 101,6 | 116,9 | 135,1 | 150,1 | 175,5 | 152,3 | 261,6 |
| Ergonomic | 43,2 | 70,7 | 96,9 | 121,9 | 121,1 | 131 | 144,1 | 170,5 | 135,7 | 266,1 |
| P-Value  Professionals | 0,445 | 0,799 | 0,721 | 0,646 | 0,878 | 0,799 | 0,575 | 0,285 | 0,241 | 0,799 |
|  |  |  |  |  |  |  |  |  |  |  |
| Standard | 60,9 | 96 | 71 | 95,2 | 97,8 | 120,1 | 203,6 | 279,8 | 162,6 | 350,4 |
| Ergonomic | 58,1 | 90 | 70,1 | 79,9 | 96,8 | 116,1 | 191,6 | 242,4 | 171,3 | 351,5 |
| P-Value  Amateurs | 0,422 | 0,311 | 0,701 | 0,422 | 0,701 | 0,6 | 0,463 | 0,249 | 0,382 | 0,552 |
|  |  |  |  |  |  |  |  |  |  |  |
| Standard | 61,6 | 103,1 | 66,5 | 107,6 | 115,6 | 145,2 | 182 | 212,8 | 150,1 | 291,3 |
| Ergonomic | 48,3 | 75,7 | 57,3 | 86,9 | 96,7 | 106,5 | 150,6 | 179,7 | 139,8 | 281,2 |
| P-Value  Playing Time ≤5h | 0,407 | 0,492 | 0,381 | 0,356 | 0,309 | 0,246 | 0,177 | 0,113 | 0,523 | 0,619 |
|  |  |  |  |  |  |  |  |  |  |  |
| Standard | 64,3 | 99,4 | 61,9 | 89,4 | 107 | 134,1 | 217,1 | 283 | 209 | 371,7 |
| Ergonomic | 56,7 | 95,9 | 72,9 | 94,8 | 119,5 | 141,5 | 203 | 260,1 | 202,1 | 351,6 |
| P-Value  Playing Time 5-20h | 0,959 | 0,508 | 0,386 | 0,241 | 0,093 | 0,508 | 0,575 | 0,333 | 0,799 | 0,799 |
|  |  |  |  |  |  |  |  |  |  |  |
| Standard | 62 | 103,1 | 88,8 | 110,8 | 115,6 | 130 | 182 | 212,8 | 150,1 | 243,7 |
| Ergonomic | 58,8 | 83,4 | 57,6 | 71,5 | 96,7 | 112,5 | 150,6 | 179,7 | 137 | 247,9 |
| P-Value  Playing Time >20h | 0,807 | 0,311 | 0,173 | 0,064 | 0,055 | 0,075 | 0,116 | **0,039*** | **0,016*** | 0,196 |
|  |  |  |  |  |  |  |  |  |  |  |
| Standard | 46,6 | 78,4 | 71 | 85,2 | 84,7 | 118,9 | 192,3 | 241 | 160,6 | 316,1 |
| Ergonomic | 55,8 | 80,3 | 71,2 | 109,3 | 77,9 | 97,6 | 165,9 | 220 | 167,8 | 351,5 |
| P-Value  VAS <2 | 0,499 | 0,31 | 0,31 | 0,237 | 0,398 | 0,398 | 0,735 | 1 | 0,398 | 0,091 |
|  |  |  |  |  |  |  |  |  |  |  |
| Standard | 60,6 | 96 | 71 | 98,4 | 107,3 | 125,5 | 203,5 | 247,8 | 162,6 | 331,6 |
| Ergonomic | 55,8 | 80,3 | 70,1 | 86,9 | 96,8 | 113,4 | 165,9 | 214,6 | 167,8 | 324 |
| P-Value  VAS ≥2 | 0,757 | 0,946 | 0,143 | 0,093 | 0,201 | 0,143 | 0,143 | 0,083 | 0,412 | 0,925 |
|  |  |  |  |  |  |  |  |  |  |  |
| Standard | 62 | 103,1 | 60,1 | 70,6 | 78,2 | 109,7 | 157,3 | 181,1 | 149,3 | 231,9 |
| Ergonomic | 77 | 110,9 | 53,8 | 70,6 | 91,2 | 116,1 | 150,6 | 179,7 | 145,5 | 247,9 |
| P-Value | 0,225 | 0,686 | 0,138 | 0,345 | 0,345 | 0,345 | 0,5 | 0,345 | 0,225 | 0,893 |
|  |  |  |  |  |  |  |  |  |  |  |
| **Lead Arm** | | | | | | | | | | |
| PT |  |  |  |  |  |  |  |  |  |  |
| Total cohort |  |  |  |  |  |  |  |  |  |  |
| Standard | 58,1 | 107,5 | 51,7 | 65,5 | 54,2 | 59,3 | 88,3 | 130,4 | 124 | 235,4 |
| Ergonomic | 54 | 93,7 | 53 | 67,3 | 54,5 | 58,8 | 88,5 | 112,4 | 118,7 | 253,4 |
| P-Value | **0,006*** | **0,017*** | 0,53 | 0,504 | 0,861 | 1 | 0,704 | 0,289 | 0,478 | 0,371 |
| Male |  |  |  |  |  |  |  |  |  |  |
| Standard | 56,7 | 109,4 | 55,1 | 69,6 | 54,7 | 61,7 | 90,1 | 134,8 | 133,3 | 246,9 |
| Ergonomic | 52,8 | 95,2 | 53,7 | 73,1 | 55,4 | 62,9 | 103,9 | 147,4 | 138,8 | 260,4 |
| P-Value | **0,04*** | 0,067 | 0,681 | 0,526 | 0,681 | 0,94 | 0,794 | 0,575 | 0,351 | 0,332 |
| Female |  |  |  |  |  |  |  |  |  |  |
| Standard | 62,9 | 93,4 | 39,3 | 50,2 | 44,8 | 51,3 | 87,9 | 130,4 | 109,2 | 205,6 |
| Ergonomic | 60,1 | 87,4 | 39,9 | 52,1 | 52 | 56,4 | 80,6 | 110,3 | 100,5 | 229,9 |
| P-Value | 0,074 | 0,139 | 0,721 | 0,959 | 0,878 | 0,721 | 0,203 | 0,285 | 0,959 | 0,646 |
| Professionals |  |  |  |  |  |  |  |  |  |  |
| Standard | 55,9 | 79,7 | 58,5 | 74,4 | 68 | 74 | 63,6 | 84,6 | 82,4 | 159 |
| Ergonomic | 54 | 79 | 54,5 | 78,8 | 55,4 | 68,6 | 73,9 | 100,9 | 93,1 | 219,1 |
| P-Value | 0,345 | 0,249 | 0,507 | 0,701 | 0,807 | 0,345 | 0,463 | 0,311 | 0,152 | 0,382 |
| Amateurs |  |  |  |  |  |  |  |  |  |  |
| Standard | 58,6 | 109,4 | 46,9 | 57,3 | 45,2 | 58,6 | 91,8 | 143,9 | 131 | 257,9 |
| Ergonomic | 54 | 94,5 | 49,5 | 60,8 | 50,2 | 53,4 | 94,4 | 113,6 | 137 | 262,7 |
| P-Value | **0,002*** | **0,025*** | 0,653 | 0,619 | 0,723 | 0,463 | 0,831 | 0,758 | 0,554 | 0,687 |
| Playing Time ≤5h |  |  |  |  |  |  |  |  |  |  |
| Standard | 70,5 | 116,5 | 35,8 | 42,7 | 56,4 | 61 | 92,9 | 135,9 | 118,4 | 243,6 |
| Ergonomic | 50,4 | 91,9 | 39,4 | 52,1 | 46,5 | 51 | 80,6 | 111,5 | 100,5 | 246,3 |
| P-Value | 0,169 | 0,074 | 0,386 | 0,386 | 0,386 | 0,61 | 0,203 | 0,093 | 0,721 | 0,721 |
| Playing Time 5-20h |  |  |  |  |  |  |  |  |  |  |
| Standard | 58,6 | 105,6 | 58,9 | 69,7 | 47,3 | 60 | 90,5 | 145,6 | 131 | 234,7 |
| Ergonomic | 55,2 | 94,5 | 57,3 | 78,8 | 56,8 | 68,6 | 112,1 | 153,9 | 137 | 263,4 |
| P-Value | **0,019*** | 0,221 | 0,6 | 0,972 | 0,861 | 0,972 | 0,6 | 0,861 | 0,552 | 0,422 |
| Playing Time >20h |  |  |  |  |  |  |  |  |  |  |
| Standard | 49,3 | 79 | 46,9 | 70,7 | 52,6 | 57,4 | 54 | 68,3 | 84,2 | 151,9 |
| Ergonomic | 50 | 78,6 | 53 | 66,5 | 55,3 | 57,3 | 73,9 | 100,9 | 111,2 | 250,9 |
| P-Value | 0,398 | 0,237 | 0,31 | 0,735 | 0,866 | 0,499 | 0,866 | 1 | 0,398 | 0,499 |
| VAS <2 |  |  |  |  |  |  |  |  |  |  |
| Standard | 61,6 | 109,4 | 58,5 | 69,7 | 57,4 | 63,5 | 91,8 | 127,9 | 122,2 | 235,9 |
| Ergonomic | 54 | 94,5 | 57,3 | 78,8 | 56,8 | 68,6 | 78,6 | 111,3 | 125,7 | 252,8 |
| P-Value | **0,025*** | 0,069 | 0,563 | 0,657 | 0,696 | 0,484 | 0,276 | 0,174 | 0,493 | 0,84 |
| VAS ≥2 |  |  |  |  |  |  |  |  |  |  |
| Standard | 51,5 | 88,5 | 22,8 | 28,4 | 32,3 | 36,2 | 86,1 | 145,6 | 125,9 | 234,7 |
| Ergonomic | 40,9 | 86,4 | 33,5 | 42,7 | 36,5 | 48,1 | 112,1 | 153,9 | 111,7 | 263,4 |
| P-Value | 0,08 | **0,043*** | 0,893 | 0,345 | 0,138 | 0,138 | 0,225 | 0,5 | 0,686 | 0,08 |
| **Trail Arm** | | | | | | | | | | |
| PT |  |  |  |  |  |  |  |  |  |  |
| Total cohort |  |  |  |  |  |  |  |  |  |  |
| Standard | 48,6 | 120,7 | 39,3 | 57,5 | 41,3 | 52,6 | 91,3 | 131,9 | 105,1 | 245,6 |
| Ergonomic | 49,6 | 105,5 | 37,7 | 52,9 | 38,7 | 47,7 | 79,9 | 117 | 100 | 244,4 |
| P-Value  Male | 0,943 | 0,845 | **0,006*** | **0,035*** | **0,021*** | **0,003*** | **0,007*** | **<0,001*** | 0,106 | 0,318 |
|  |  |  |  |  |  |  |  |  |  |  |
| Standard | 48,6 | 124,5 | 41,2 | 67,7 | 39,1 | 47,5 | 91,3 | 134,6 | 109,1 | 245,6 |
| Ergonomic | 53,9 | 123,4 | 38,6 | 59,1 | 33,9 | 40 | 79,2 | 118,8 | 102 | 255 |
| P-Value  Female | 0,526 | 0,55 | **0,044*** | **0,019*** | 0,218 | **0,033*** | 0,073 | **0,017*** | 0,37 | 0,794 |
|  |  |  |  |  |  |  |  |  |  |  |
| Standard | 46,5 | 84,9 | 35,3 | 45,8 | 54,5 | 65 | 91,1 | 126,8 | 102,1 | 230,2 |
| Ergonomic | 44,5 | 82,5 | 33,2 | 40,5 | 42,8 | 52,9 | 85,8 | 110,3 | 82 | 207,3 |
| P-Value  Professionals | 0,333 | 0,646 | **0,037*** | 0,799 | **0,022*** | **0,047*** | **0,022*** | **0,013*** | 0,093 | 0,093 |
|  |  |  |  |  |  |  |  |  |  |  |
| Standard | 46,9 | 120,6 | 33 | 51 | 34,2 | 45,5 | 73,6 | 118,3 | 90,1 | 219,8 |
| Ergonomic | 47,5 | 97,8 | 32,2 | 47,5 | 32,9 | 37,1 | 79 | 109,2 | 100,5 | 257,3 |
| P-Value  Amateurs | 0,972 | 0,861 | 0,116 | 0,101 | 0,101 | **0,039*** | 0,152 | **0,046*** | 0,972 | 0,701 |
|  |  |  |  |  |  |  |  |  |  |  |
| Standard | 50,5 | 120,8 | 44,2 | 57,7 | 52,5 | 64,9 | 100,4 | 143,7 | 106,7 | 271,9 |
| Ergonomic | 51,7 | 113,3 | 38,5 | 55 | 42,8 | 50,7 | 86,7 | 120,9 | 99 | 230,5 |
| P-Value  Playing Time ≤5h | 0,943 | 0,981 | **0,025*** | 0,124 | 0,076 | **0,039*** | **0,022*** | **0,009*** | **0,044*** | 0,068 |
|  |  |  |  |  |  |  |  |  |  |  |
| Standard | 48 | 104,5 | 30,4 | 41,6 | 40,5 | 47,8 | 86,7 | 128,9 | 105,1 | 272,8 |
| Ergonomic | 45,4 | 98,3 | 25,7 | 38,9 | 34,4 | 40,6 | 74,2 | 107,6 | 84,2 | 230,1 |
| P-Value  Playing Time 5-20h | 0,575 | 0,575 | **0,005*** | 0,508 | **0,028*** | **0,017*** | **0,007*** | **0,005*** | **0,007*** | 0,059 |
|  |  |  |  |  |  |  |  |  |  |  |
| Standard | 50,5 | 120,8 | 46 | 66,1 | 54,7 | 65,4 | 110,3 | 158,9 | 128,9 | 236,6 |
| Ergonomic | 53,4 | 113,3 | 41,9 | 55 | 57,7 | 61,4 | 108,9 | 145,1 | 135,6 | 276,4 |
| P-Value  Playing Time >20h | **0,046*** | 0,382 | 0,382 | 0,101 | 0,221 | 0,064 | 0,463 | 0,173 | 0,422 | 0,279 |
|  |  |  |  |  |  |  |  |  |  |  |
| Standard | 46,9 | 120,6 | 33 | 61,9 | 30,5 | 34,5 | 62,8 | 93,7 | 100,9 | 204 |
| Ergonomic | 35,9 | 83,9 | 32,2 | 47,5 | 32,9 | 37,1 | 73,9 | 109,2 | 99 | 210,3 |
| P-Value  VAS <2 | 0,398 | 0,735 | 0,612 | 0,398 | 0,612 | 0,31 | 0,128 | 0,091 | 0,176 | 0,398 |
|  |  |  |  |  |  |  |  |  |  |  |
| Standard | 50,3 | 128,2 | 42,6 | 61,9 | 41,4 | 55 | 93,7 | 134,8 | 111,6 | 255,1 |
| Ergonomic | 51,9 | 118,1 | 39,3 | 58,7 | 40,5 | 48,5 | 80,3 | 118,7 | 100,5 | 257,3 |
| P-Value  VAS ≥2 | 0,778 | 0,925 | **0,007*** | **0,025*** | **0,015*** | **0,002*** | **0,004*** | **<0,001*** | 0,201 | 0,459 |
|  |  |  |  |  |  |  |  |  |  |  |
| Standard | 42,4 | 73,2 | 14,7 | 17,8 | 23 | 35,4 | 67,3 | 117,5 | 93,4 | 188,6 |
| Ergonomic | 43,2 | 78,6 | 13,9 | 22 | 19,7 | 25,7 | 50,6 | 84,4 | 72,6 | 179,7 |
| P-Value | 0,345 | 0,5 | 0,345 | 0,686 | 0,893 | 0,893 | 0,893 | 0,5 | 0,345 | 0,345 |
| **Lead Arm** | | | | | | | | | | |
| BB |  |  |  |  |  |  |  |  |  |  |
| Total cohort |  |  |  |  |  |  |  |  |  |  |
| Standard | 13,9 | 25,4 | 21,6 | 29,6 | 34,9 | 43,4 | 77,8 | 142 | 155,7 | 276 |
| Ergonomic | 14,1 | 31,3 | 20,5 | 30,4 | 35,7 | 49,5 | 86,3 | 149,9 | 163,9 | 277,6 |
| P-Value  Male | 0,465 | 0,294 | 0,206 | 0,417 | 0,057 | 0,159 | 0,441 | 0,688 | 0,845 | 0,309 |
|  |  |  |  |  |  |  |  |  |  |  |
| Standard | 12,7 | 24,2 | 19,2 | 32,4 | 42,6 | 50,2 | 84,9 | 144,2 | 172,1 | 302 |
| Ergonomic | 14,1 | 33,4 | 21,9 | 30,4 | 41,5 | 54,9 | 90,7 | 158,2 | 178 | 301,1 |
| P-Value  Female | 0,296 | 0,232 | 0,156 | 0,433 | 0,156 | 0,478 | 0,709 | 0,654 | 0,881 | 0,911 |
|  |  |  |  |  |  |  |  |  |  |  |
| Standard | 16,7 | 25,4 | 21,6 | 28,1 | 26,9 | 30,3 | 67,1 | 121 | 118,4 | 207,4 |
| Ergonomic | 17,5 | 28,9 | 19,5 | 28,2 | 30,9 | 36,5 | 73,8 | 137 | 117,4 | 246,2 |
| P-Value  Professionals | 0,721 | 0,838 | 0,878 | 0,799 | 0,241 | 0,169 | 0,646 | 0,959 | 0,799 | 0,169 |
|  |  |  |  |  |  |  |  |  |  |  |
| Standard | 12,8 | 26,2 | 22,5 | 44 | 42,5 | 44,4 | 82,9 | 134,9 | 158,2 | 251,2 |
| Ergonomic | 12,6 | 35,7 | 26,5 | 43,7 | 54,6 | 57,4 | 89,1 | 157,6 | 157,8 | 282,3 |
| P-Value  Amateurs | 0,753 | 0,422 | 0,345 | 0,972 | 0,463 | 0,917 | 0,861 | 0,311 | 0,972 | 0,753 |
|  |  |  |  |  |  |  |  |  |  |  |
| Standard | 14,5 | 24,6 | 19,3 | 27,1 | 29,3 | 32 | 76,8 | 143,5 | 151,4 | 281,3 |
| Ergonomic | 14,6 | 31,2 | 20,3 | 27,8 | 34,8 | 46,2 | 83,6 | 144 | 170 | 272,9 |
| P-Value  Playing Time ≤5h | 0,523 | 0,478 | 0,356 | 0,266 | 0,084 | 0,093 | 0,227 | 0,795 | 0,795 | 0,287 |
|  |  |  |  |  |  |  |  |  |  |  |
| Standard | 14 | 27,7 | 20,4 | 35,7 | 56,3 | 68,1 | 114,6 | 164,8 | 156,9 | 302,8 |
| Ergonomic | 17,8 | 35,9 | 20,4 | 31,5 | 46,5 | 58,7 | 113,9 | 149,9 | 157,8 | 287,2 |
| P-Value  Playing Time 5-20h | 0,646 | 0,386 | 0,386 | 0,169 | 0,169 | 0,285 | 0,386 | 0,169 | 0,575 | 0,203 |
|  |  |  |  |  |  |  |  |  |  |  |
| Standard | 12,5 | 24,6 | 19,3 | 26,8 | 31,7 | 44,2 | 73,6 | 138 | 165,5 | 281,3 |
| Ergonomic | 12,6 | 31,2 | 20,3 | 27,8 | 34,8 | 52,8 | 83,6 | 157,6 | 170 | 282,3 |
| P-Value  Playing Time >20h | 0,753 | 0,6 | 0,249 | 0,552 | 0,055 | 0,075 | **0,046**** | 0,382 | 0,917 | 0,753 |
|  |  |  |  |  |  |  |  |  |  |  |
| Standard | 16,1 | 26,2 | 24,4 | 36,9 | 27,2 | 33,6 | 65 | 100,3 | 153,2 | 239,2 |
| Ergonomic | 14 | 35,7 | 26,5 | 30,9 | 29,5 | 32,6 | 83,5 | 95,7 | 136,3 | 192,7 |
| P-Value  VAS <2 | 1 | 0,735 | 1 | 0,735 | 0,866 | 0,499 | 0,499 | 0,612 | 0,31 | 0,398 |
|  |  |  |  |  |  |  |  |  |  |  |
| Standard | 13,5 | 24,6 | 22,4 | 30,6 | 40,1 | 44,4 | 82,9 | 142,3 | 165,5 | 281,3 |
| Ergonomic | 14 | 31,4 | 23,2 | 32,9 | 37,7 | 54,8 | 89,1 | 155,9 | 170 | 282,3 |
| P-Value  VAS ≥2 | 0,104 | 0,065 | 0,192 | 0,353 | 0,083 | 0,231 | 0,51 | 0,84 | 0,657 | 0,135 |
|  |  |  |  |  |  |  |  |  |  |  |
| Standard | 18,9 | 45,9 | 12,6 | 19,4 | 23,8 | 28,6 | 44,8 | 73,7 | 115 | 233,5 |
| Ergonomic | 14,6 | 31,2 | 12,5 | 21,5 | 22,2 | 28,7 | 42,1 | 67,5 | 120,3 | 228,9 |
| P-Value | **0,043*** | 0,08 | 0,686 | 0,893 | 0,225 | 0,345 | 0,686 | 0,345 | 0,686 | 0,138 |
| **Trail Arm** | | | | | | | | | | |
| BB |  |  |  |  |  |  |  |  |  |  |
| Total cohort |  |  |  |  |  |  |  |  |  |  |
| Standard | 40,1 | 98,1 | 38,2 | 57,3 | 41,7 | 47,7 | 66,8 | 137,6 | 125,7 | 300,5 |
| Ergonomic | 37,4 | 95,2 | 35,7 | 53,2 | 40 | 43,5 | 73,8 | 118,4 | 131,5 | 326,2 |
| P-Value  Male | 0,185 | 0,544 | 0,894 | 0,237 | 0,371 | 0,453 | 0,877 | 0,202 | 0,734 | **0,047**** |
|  |  |  |  |  |  |  |  |  |  |  |
| Standard | 40,1 | 93,9 | 43,1 | 61,4 | 43,4 | 50,9 | 83,5 | 147,4 | 137,3 | 329,9 |
| Ergonomic | 39,5 | 103,2 | 54,1 | 62,5 | 40 | 43,5 | 87 | 130,2 | 157,1 | 335,2 |
| P-Value  Female | 0,654 | 0,94 | 0,881 | 0,502 | 0,411 | 0,654 | 0,852 | 0,145 | 0,627 | 0,279 |
|  |  |  |  |  |  |  |  |  |  |  |
| Standard | 39 | 103,2 | 14,3 | 42,8 | 31,1 | 33,2 | 61,9 | 121,4 | 102,1 | 268,5 |
| Ergonomic | 34,7 | 89 | 26,8 | 36,9 | 39,2 | 45 | 68,9 | 117,5 | 106 | 285 |
| P-Value  Professionals | **0,047*** | 0,333 | 0,646 | 0,203 | 0,878 | 0,386 | 0,878 | 0,76 | 0,241 | 0,059 |
|  |  |  |  |  |  |  |  |  |  |  |
| Standard | 36,5 | 80,6 | 31,9 | 56,4 | 30,2 | 32,7 | 41,6 | 57,9 | 93,4 | 293,3 |
| Ergonomic | 31,4 | 80,8 | 25,8 | 53,2 | 35,1 | 39,3 | 49,5 | 52,6 | 93,4 | 253,1 |
| P-Value  Amateurs | 0,173 | 0,972 | 0,552 | 0,552 | 0,116 | 0,116 | 0,701 | 0,861 | 0,917 | 0,249 |
|  |  |  |  |  |  |  |  |  |  |  |
| Standard | 43 | 122,6 | 39,5 | 67,3 | 48,1 | 52,2 | 75,4 | 142,9 | 131 | 307,7 |
| Ergonomic | 49,6 | 115,2 | 53,8 | 63,4 | 47,2 | 51,3 | 91,7 | 135 | 141,7 | 338,8 |
| P-Value  Playing Time ≤5h | 0,653 | 0,381 | 0,407 | 0,287 | 0,906 | 0,831 | 0,653 | 0,136 | 0,687 | 0,102 |
|  |  |  |  |  |  |  |  |  |  |  |
| Standard | 39 | 109,8 | 49,6 | 63,4 | 48,7 | 55,7 | 93,6 | 147,5 | 125,7 | 320,9 |
| Ergonomic | 37,4 | 86,1 | 51 | 60,6 | 47,4 | 54,9 | 97,7 | 124,5 | 139,9 | 335,2 |
| P-Value  Playing Time 5-20h | 0,285 | 0,203 | 0,139 | **0,013*** | 0,878 | 0,878 | 0,386 | 0,169 | 0,799 | 0,203 |
|  |  |  |  |  |  |  |  |  |  |  |
| Standard | 43,5 | 99,9 | 37,4 | 56,4 | 40,4 | 45,2 | 61,5 | 115 | 101,6 | 265,1 |
| Ergonomic | 47,5 | 103,4 | 33,2 | 51,9 | 43,2 | 51,1 | 68,2 | 113,1 | 128,9 | 304 |
| P-Value  Playing Time >20h | 0,6 | 0,65 | 0,422 | 0,701 | **0,039**** | 0,101 | 0,463 | 0,917 | 0,917 | 0,196 |
|  |  |  |  |  |  |  |  |  |  |  |
| Standard | 11,9 | 42,1 | 31,9 | 44,7 | 30,2 | 32,7 | 38,1 | 54,3 | 134,8 | 315,9 |
| Ergonomic | 15,8 | 37,8 | 24,7 | 34 | 35,1 | 39 | 37,4 | 40,3 | 98,6 | 253,1 |
| P-Value  VAS <2 | 0,612 | 0,735 | 1 | 0,398 | 0,31 | 0,398 | 0,735 | 0,612 | 0,612 | 0,31 |
|  |  |  |  |  |  |  |  |  |  |  |
| Standard | 41 | 99,2 | 39,5 | 59,6 | 43 | 50,2 | 64,9 | 140,3 | 120,4 | 307,7 |
| Ergonomic | 37,4 | 101,1 | 48,2 | 57,9 | 42,2 | 45,2 | 72,2 | 111 | 137,3 | 331,6 |
| P-Value  VAS ≥2 | 0,253 | 0,459 | 0,968 | 0,242 | 0,427 | 0,313 | 0,619 | 0,706 | 0,253 | **0,016**** |
|  |  |  |  |  |  |  |  |  |  |  |
| Standard | 35,9 | 72,5 | 27,2 | 37,1 | 28,2 | 30 | 75,4 | 134,9 | 133,9 | 293,3 |
| Ergonomic | 37,4 | 80,8 | 28,9 | 51,9 | 31,4 | 34 | 75,4 | 123,6 | 109,5 | 266 |
| P-Value | 0,686 | 0,893 | 0,893 | 0,686 | 0,5 | 0,893 | 0,08 | **0,043*** | 0,08 | 0,5 |
|  |  |  |  |  |  |  |  |  |  |  |

**Table 1.** Results of the subgroup analysis.

Mean and maximum µV of assessed muscle activity during the five phases of the golf swing.

Statistically significant p-values are shown in bold, * indicates a statistically significant decrease in muscle activity with the ergonomic grip, ** indicates a statistically significant increase in muscle activity with the ergonomic grip.

VAS: Visual analogue scale
